# Major discrepancies in recommendations regarding long-term activity restrictions following knee replacement: a survey among Swedish physiotherapists

**DOI:** 10.1186/s12891-026-10142-2

**Published:** 2026-07-04

**Authors:** Kristin Gustafsson, Thérése Jönsson, Marcus Ljung, Caroline Ståhl, Elin Östlind

**Affiliations:** 1 Department of Health, Medicine and Caring Sciences, Unit of Physiotherapy, Linköping University, Linköping, Sweden; 2 Rehabilitation Centre, Ryhov County Hospital, Jönköping, Sweden; 3 Department of Health Sciences, Faculty of Medicine, Lund University, Lund, Sweden; 4 Department of Orthopaedics, Skåne University Hospital, Lund, Sweden; 5 Department of Orthopaedics, Rehabilitation Unit, Vrinnevi County Hospital, Norrköping, Sweden; 6 Department of Orthopaedics, Ängelholm Hospital, Ängelholm, Sweden; 7 Dalby Healthcare Centre, Dalby, Sweden

**Keywords:** Knee Replacement, Physiotherapy, Recommendations, Physical activity

## Abstract

**Background:**

There is currently limited evidence to support specific recommendations for long-term activity restrictions after knee replacement surgery. Consequently, physiotherapists´ guidance may vary, which could influence patients´ confidence, adherence to rehabilitation, overall recovery outcomes, and even contribute to the development of fear of movement. However, it is unclear to what extent such variation exists. This study aimed to explore and describe the recommendations given by physiotherapists regarding long-term activity restrictions following knee replacement.

**Methods:**

An online survey was distributed to physiotherapy units in Swedish orthopedic specialist clinics and primary healthcare centers. For this study, responses to the question “Do you usually recommend any long-term restrictions? (For example, running, skiing, squatting, kneeling?) Please elaborate!” were analyzed using quantitative content analysis to systematically identify and quantify patterns.

**Results:**

Of 233 physiotherapists who responded to the specific survey question, 66% (*n* = 151) reported that they provided specific recommendations regarding long-term activity restrictions following knee replacement. The most commonly non-recommended activities were running and jumping, followed by kneeling and squatting. However, the extent of these restrictions varied considerably, ranging from complete avoidance of certain activities to conditional approval based on intensity and frequency. Among those recommending long-term restrictions, 16% (*n* = 24) stated that the primary reason was to avoid compromising implant survival.

**Conclusions:**

Considerable variability exists in physiotherapists´ recommendations regarding long-term activity restriction following knee replacement across both orthopedic clinics and primary healthcare settings. This variation reflects the current lack of evidence-based guidelines and highlights the need for consensus to support more consistent and confident communication with patients about long-term physical activity.

## Background

Knee osteoarthritis (OA) is a leading cause of pain and functional limitation in weight-bearing activities such as sports, walking, and manual labor among middle-aged and older [1]. First-line treatment includes physical activity, patient education, and weight control if necessary [2–4]. If insufficient, additional options such as analgesics and walking aids may be considered [1, 2, 4]. For patients with persistent symptoms and substantially reduced quality of life, a total or partial knee replacement (KR) may be indicated [2]. KR is the most frequently performed orthopedic surgery globally [5], with approximately 20,000 primary surgeries performed annually in Sweden [6].

Rehabilitation after KR should be structured and coordinated to optimize outcomes, focusing on range of motion (ROM) muscle strength, physical function and quality of life [7]. A clearly defined rehabilitation strategy facilitates patients´ return to an independent and active lifestyle [8, 9]. Physiotherapists (PTs) initiate early mobilization and functional exercises in hospitals, with continued rehabilitation in primary healthcare. Recovery is often prolonged and challenging [10, 11], with major gains in ROM typically achieved within three months, while restoration of muscle strength symmetry may take over a year [12].

Traditionally, patients undergoing KR surgery have been advised to avoid life-long participation in activities such as running, jumping, kneeling and heavy lifting, based on the assumption that mechanical stress may contribute to implant wear and increase the risk of revision surgery [13]. Existing international recommendations regarding long-term physical activity after KR have largely been shaped by clinical experience and survey data rather than robust empirical evidence, and have primarily focused on total rather than partial KRs [14, 15]. Recent research has begun to question these traditional assumptions, suggesting that higher levels of physical activity and sports participation may not necessarily compromise implant survival, even in the longer term, with no increased risk of revision surgery observed for up to twelve years postoperatively [16–18]. This is of particular relevance given the increasing prevalence of KR among patients younger than 55 years of age [19], who often have higher expectations of maintaining an active lifestyle.

Despite these findings and the growing number of younger, more active patients, there is still a lack of consensus to provide recommendations regarding long-term return to higher physical activity after KR [20]. The absence of clear, evidence-based guidelines may contribute to variability and inconsistency in the advice provided to patients by healthcare professionals [14, 18, 21]. Furthermore, little is known about the specific guidance clinicians’ offer and how it is applied in practice.

To support future guideline development and promote more consistent patient-centered care, a better understanding of current clinical practices is needed. Therefore, the aim of this study was to explore and describe the recommendations provided by PTs regarding long-term activity restrictions following KR.

## Methods

### Study design and setting

This cross-sectional survey study was conducted in physiotherapy practice at Sweden. In accordance with national ethical guidelines, formal ethical approval was not required, as the study did not involve the collection of personal or identifiable data. The study was reported according to the consensus-based checklist for reporting of survey studies (the CROSS checklist) [22]. Microsoft Copilot was used for language editing and was not used in any other aspects of the research process.

In Sweden, the healthcare system is organized into 21 self-governing regions, each responsible for planning, managing, financing and prioritizing public or private healthcare services. Physiotherapy is provided across the care continuum based on patients’ need. In specialized care, physiotherapy is focused on assessment, early mobilization after surgery or acute illness, therapeutic interventions and coordination of continued rehabilitation – through either inpatient care or specialized outpatient follow-ups. In primary healthcare, PTs offer a broad range of assessment, treatment and preventive interventions for patients with various conditions. Patients may access these services via referral from specialized care or general practitioners, or through direct contact with a healthcare centre or physiotherapy unit. Healthcare is mainly tax-funded, resulting in low individual costs [23].

In response to the high prevalence of KR in Sweden, an interdisciplinary national working group was established in 2023, to design a cohesive and patient-centered clinical healthcare pathway for patients undergoing KR [24]. To gain information regarding current postoperative rehabilitation, a nationwide survey was conducted to gather information from PTs about current rehabilitation practices for patients undergoing KR.

### Survey development

Three members of the working group – EÖ, ML and CS – all experienced PTs in knee replacement rehabilitation, designed the online survey. It included 15 main questions covering rehabilitation practice, treatment modalities and recommendations to patients regarding long-term activity. This study focused specifically on the survey question addressing recommendations for long-term physical activity restrictions following KR. The survey was administered using the Sunet Survey platform [25]. Prior to data collection, a pilot version was tested with five primary care PTs, resulting in minor adjustments based on their feedback. No personal information was collected from respondents, apart from region and their type of workplace. Further details regarding the survey´s development, distribution and content have been reported previously [26].

### Data collection and participants

The online survey was distributed as a public open survey to PTs at all 80 orthopedic clinics in Sweden performing KRs, identified through the Swedish Arthroplasty Register, which covers 97–98% of all KRs performed in the country [27]. It was also shared with PTs working in primary healthcare who treat patients postoperatively, via the Swedish Physiotherapy Association, social media, and professional networks with PTs across different regions. At each unit, participating PTs were asked to complete the survey collectively, reflecting their local and regional guidelines and practices for KR rehabilitation. Completion of the survey was considered as providing written informed consent to participate. The survey was open from November 2023 to January 2024; however, due to its public and anonymous nature, no reminders were sent.

### Data analysis

To address the study´s aim of examining physiotherapists’ recommendations on long-term activity restrictions after KR, we analyzed responses to the following open-ended survey question: *“Do you usually recommend any long-term restrictions? (For example, running, skiing, squatting, kneeling?) Please elaborate.”* We used quantitative content analysis, a systematic method for objectively identifying and quantifying specific elements within textual material. This approach involves defining clear coding categories, applying them consistently, and quantifying the results to reveal patterns and distributions [28].

The research objective was initially defined collaboratively by all authors. Two authors, KG and EÖ, both experienced in quantitative and qualitative methodology, and in KR rehabilitation in specialized care and primary healthcare settings, were primarily responsible for the analysis, with input from the other co-authors. They first reviewed all responses to gain a comprehensive understanding of the material. Free-text responses relevant to the study aim were identified, and condensed into codes that reflected the core content of each response. These codes were then sorted into categories [28, 29].

KG and EÖ performed the coding and categorization independently to assess inter-coder reliability and ensure consistency [28]. Following this, TJ, KG and EÖ discussed and revised the codes and categories until consensus was reached, ensuring that the categories were mutually exclusive and exhaustive. Finally, the coded data were summarized using descriptive statistics (frequencies and percentages) to identify patterns within the data. The analysis and the data organization were conducted using Microsoft Excel. Results are presented both in text and as the number of responses within each category, supported by illustrative quotes.

## Results

A total of 233 PTs participated in the survey. Of these, 230 provided responses relevant to the study´s aim. Among them, 38 worked at orthopedic clinics, representing 50% of the clinics to which the survey was distributed. In the primary healthcare sector, 192 PTs from various units responded, with representation from 18 out of Sweden´s 21 healthcare regions.

**Fig. 1:**
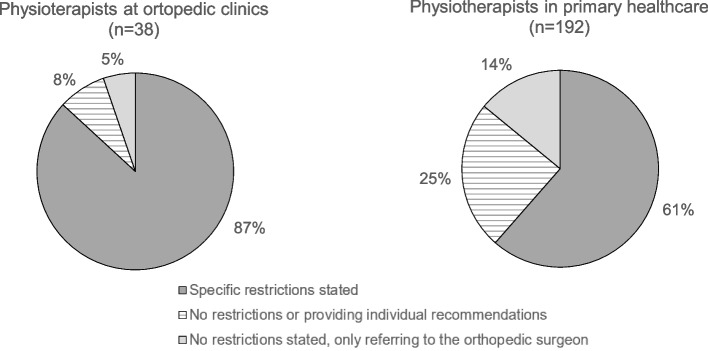
Distribution of physiotherapists´ approaches to long-term activity restrictions following knee replacement surgery

The majority of participants (66%, *n* = 151) reported providing specific recommendations regarding long-term activity restrictions after KR – 87% (*n* = 33) of PTs in orthopedic clinics and 61% (*n* = 118) in primary healthcare. In contrast, 22% of all PTs (*n* = 50), stated that they either did not provide restrictions or offered individualized advice. Additionally, 13% (*n* = 29) referred patients to the orthopedic surgeon for guidance. PTs in orthopedic clinics were more likely to provide specific recommendations, while those in primary care more often referred patients to the surgeon (14% vs. 5%) (Fig. 1).

*We do not have any general recommendations regarding this [long-term restrictions]; instead, the recommendations may vary depending on the surgeon. We are usually aware of how the different surgeons think and adapt accordingly. However, we sometimes refer directly to the surgeon when questions arise about specific activities.* (PT25 at an orthopedic clinic).

### Recommendations regarding specific long-term activity restrictions

Among the 151 PTs who reported providing recommendations on long-term activity restrictions, the majority specified particular activities to avoid. **Running** emerged as the most frequently non-recommended activity. However, the nature and extent of this restriction varied considerably – ranging from strict avoidance to conditional approval, depending on factors such as intensity, frequency and patient context. PTs working in orthopedic clinics were more likely to advice against running than those in primary care. The most common responses were brief statements and directive, such as “*No running”* or “*We advise against running.”* Others offered more nuanced explanations, highlighting the intent behind the recommendations:

*….Running is also not recommended, but it is not forbidden, i.e., they can run or jog to catch a bus, but not engage in regular running for recreational races…. We inform patients that the primary aim of the surgery is to relieve pain - not to become a better runner.* (PT71 in primary healthcare).

**Jumping** and other **impact-loading activities**, such as dancing, were the second most commonly non-recommended activity. Many PTs used firm language like “*Avoid jumping – for life”* or “*No jumping or impact-loading activities.”* However, some provided more reflective reasoning, balancing clinical caution with patient-centered care:

*I usually recommend that they avoid running long distances or doing a lot of jumping. But most don´t have a high activity level to begin with, and in those cases, my experience is that it is better not to make them afraid of movement* (PT24 in primary healthcare).

**Kneeling** was also mentioned as a non-recommended activity. While most PTs did not elaborate on their rationale, a few noted that kneeling could cause discomfort or pain. Some suggested it might be acceptable if performed on a soft surface. Similarly, **squatting** was frequently not recommended, though several PTs remarked that this recommendation often had limited practical relevance, as many patients found it physically difficult to perform a full squat after KR.

Fewer responses addressed **high-impact or explosive sport**, but when they did, a wide range of terms were used – such as *intensive training, high-speed activities, contact sports, pivoting activities* and *extreme sports*. Specific examples included down-hill skiing, parachuting, motor cross and cross-country running.

**Fig. 2:**
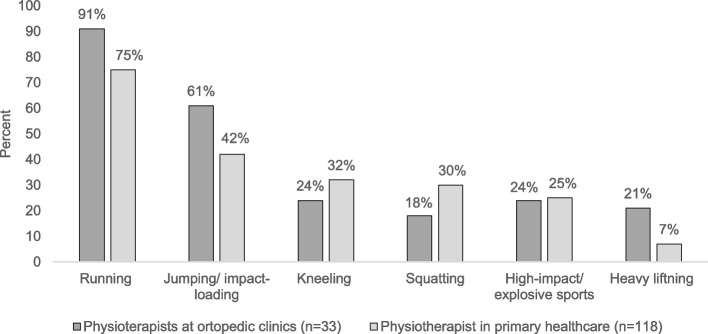
Proportion of non-recommended activities following knee replacement from physiotherapists (PTs) working in orthopedic clinics and primary healthcare settings. Data are presented as percentages based on the numbers of PTs in each group who indicated that they provide specific recommendations on long-term activity restriction

In some cases, **heavy lifting** was also identified as a non-recommended activity. Recommendations varied, with some PTs advising patients to avoid lifting or carrying loads exceeding a specific weight – typically between 15 and 25 kg – while others recommended avoiding from heavy lifting altogether (Fig. 2).

*Do not carry heavy loads, about 20 kg. Works occasionally but repeated lifting every day as with heavy work should be avoided.* (PT18 in primary healthcare).

### Rationale for physiotherapists’ recommendations

Several participants elaborated on the reasoning behind their long-term activity recommendation following KR. The most frequently cited rationale for imposing restrictions was to increase the survival of the implant. According to the PTs, patients were advised to avoid specific activities that could place excessive mechanical stress on the joint or accelerate prosthetic wear. Among the 151 PTs who reported providing long-term activity recommendations, 24% of those worked in orthopedic clinics and 14% in primary healthcare, stated that the primary purpose of these restrictions was to enhance survival of the implant.

*Yes, downhill skiing, running and kneeling. These [recommendations] are [provided] in the light of the importance that the patient should be aware that such activities affect the lifespan of the prosthesis* (PT21 at an orthopedic clinic).

In addition to concerns absolute implant wear, PTs also expressed assumptions that high-impact or pivoting movements could increase the risk of falls and periprosthetic fractures – particularly in patients with reduced balance or muscle strength. This reasoning was reflected in the responses of eight PTs who emphasized fall risk as a rationale for recommending activity restrictions, among them 3 PTs (9%) from orthopedic clinics and 5 PTs (4%) from primary healthcare.

*I recommended avoiding or being cautious with high-speed sports, as a fall carries a significant risk of bone fracture at the site of the prosthesis (*PT108 in primary healthcare).

## Discussion

This study explored how PTs reason about and formulate long-term activity recommendations following KR. The findings reveal considerable variability in the advice provided by PTs in Sweden. The provided recommendations appear to be shaped more by clinical experience, perceived risks, and assumptions about implant survival, than by evidence-based guidelines, an approach that may contribute to inconsistencies in postoperative care and uncertainty among both clinicians and patients.

A central observation was that running, jumping, and other impact-loading activities – such as dance – were frequently non-recommended, although the degree of restriction varied. Many PTs advised against running altogether, while others permitted limited running under specific conditions, such as short distances on soft surfaces. Similar variation was observed in recommendations regarding jumping and other impact-loading activities, as well as several activities commonly classified as high-impact activities, such as downhill skiing. While some PTs explicitly advised against such activities, others considered them acceptable depending on individual factors, such as the patient´s previous experience or skill level. These variations reflect a broader uncertainty in postoperative management following KR, likely influenced by limited availability in robust evidence and the absence of clear guidance on appropriate recommendations for returning to recreational activities and sports [20].

At present, there are no unified or comprehensive guidelines for activity recommendations following KR; instead existing recommendations often differ in scope and focus. In general, current guidelines are largely based on contributing physicians’ clinical experience and survey data [14, 20] and suggest that patients may resume low- to moderate-impact activities such as walking, cycling on level ground, skiing and weightlifting after surgery. However, guidance becomes less clear regarding high-impact activities such as running, cross-country skiing, and contact sports. Although these activities are sometimes included in the recommendations, most contributing physicians refrain from taking a definitive stance. Instead, uncertainty is commonly expressed, or decisions are suggested to be made on an individual basis in consultation with the patient [14, 20].

In daily clinical practice, comparing published recommendations can therefore be challenging due to differences in focus. For example, Straat et al. [21], examined activities of daily living, whereas Thaler et al. [14] focused on sports participation. This lack of consistency may contribute to ambiguity, particularly for PTs, who are often the professionals most closely involved in guiding patients through rehabilitation. In the absence of more concrete and coherent guidance, PTs may feel hesitant to make firm, individualized recommendations regarding when – or whether – certain activities, especially high-impact ones, can be safely resumed.

This uncertainty regarding which recommendations to provide is particularly notable given that recent research has not demonstrated an increased risk for revision surgery among patients who engage in higher levels of physical activity [16–18]. For example, Kornuijt et al. [17] and Crawford et al. [16] found no association between higher activity levels and revision rates following total KRs, while Teoli et al. [18] reported similar findings for partial KRs. These findings challenge the commonly held belief that high-impact activities accelerate prosthetic wear and increase the risk of implant failure.

Nevertheless, 16% of the PTs who reported recommending activity restrictions after KR did so based on the assumption that limiting certain activities would reduce prosthetic wear and extend implant lifespan – despite a lack of consensus in which activities should be restrict. This perspective is also shared by many orthopedic surgeons, who cite concerns about aseptic loosening and polyethylene wear [14]. However, the overall revision rate at 10 years remains low (approximately 3–5%), with implant loosening, infection, pain and instability as the most common causes [5].

Building on the observed variability in postoperative recommendations, this inconsistency appears even more pronounced in primary healthcare settings. Many PTs report referring patients back to the orthopedic surgeon for activity-related questions. While this approach aims to ensure alignment with the individual´s surgical protocol, it can be problematic. Surgeons are often difficult to reach, which may delay guidance, prolong rehabilitation, and increase patient uncertainty.

Similarly, PTs working in orthopedic clinics described providing activity restrictions, but the content and scope of these recommendations varied considerably. These findings align with those of Straat et al. [21], who analyzed patient information materials from Dutch hospitals and found substantial inconsistencies in the types of activities addressed and the timelines for resumption. Together, these results points to a lack of consistent, evidence-based guidance to support clinicians and patients navigating activity decisions after KR [30].

As implants technology evolves and earlier concerns about high physical activity levels are increasingly challenged [16–18], there is a growing need for updated, individualized guidelines [30]. KRs are now more frequently performed in younger patients [19], who often have higher expectations for return to active lifestyles. Although most patients report favorable outcomes, 10–20% remain dissatisfied [5, 31] – particular younger individuals, for whom unmet expectations about returning to demanding activities are common [31]. The variations in recommendations identified in this study may have important consequences for the individual patients and are therefore highly relevant to highlight to stakeholders involved in the decision-making and guideline development, as well as to clinicians, including PTs and orthopedic surgeons. Our findings reveal substantial discrepancies in recommendations regarding long-term activity restrictions following KRs, underscoring the need for tailored and realistic guidance that considers factors such as age, body compositions, occupational demands, and prior experience with high-impact activities [21]. Overly cautious or outdated recommendations may discourage patients from resuming desired activities and contribute to fear of movement – particularly when repeated warnings about implant damage reinforce avoidance behaviors, which may negatively affect recovery and well-being [32].

### Limitations

Some limitations of the study should be acknowledged. The survey question included examples of activities — running, skiing, squatting and kneeling — which may have influenced the participants’ responses. However, the frequent mention of “jumping/impact-loading activities”, which were not explicitly listed, suggests that PTs also considered activities beyond those prompted. Another limitation is the lack of distinction in the question between recommendations for partial and total KRs, an important differentiation in the literature [18, 20]. Finally, as the survey was distributed as a public, open survey, an exact response rate could not be determined, particularly for PTs in primary healthcare. Self-selection bias may have influenced the responses, as participation was voluntary.

## Conclusions

This study demonstrates considerable variability in physiotherapists’ recommendations regarding long-term activity restrictions following KR, across both orthopedic clinics and primary healthcare settings. Running was the most frequently reported non-recommended activity, followed by jumping or other impact-loading activities. These findings underscore the need for updated, evidence-based consensus on activity recommendations after KRs in order to reduce clinical uncertainty and promote consistent, patient-centered rehabilitation aligned with current research.

## Data Availability

The dataset used and analysed during the current study are available from the corresponding author on reasonable request.

## Abbreviations

OA: Osteoarthritis
KR: Knee replacement
ROM: Range of motion
PT: Physiotherapist
